# Effects of Overexpression of *WRI1* and Hemoglobin Genes on the Seed Oil Content of *Lepidium campestre*

**DOI:** 10.3389/fpls.2016.02032

**Published:** 2017-01-09

**Authors:** Emelie Ivarson, Nélida Leiva-Eriksson, Annelie Ahlman, Selvaraju Kanagarajan, Leif Bülow, Li-Hua Zhu

**Affiliations:** ^1^Department of Plant Breeding, Swedish University of Agricultural SciencesAlnarp, Sweden; ^2^Department of Pure and Applied Biochemistry, Lund UniversityLund, Sweden

**Keywords:** *Lepidium campestre*, metabolic engineering, hemoglobin, *WRINKLED1*, oil content

## Abstract

The wild species field cress (*Lepidium campestre*), belonging to the Brassicaceae family, has potential to be developed into a novel oilseed- and catch crop, however, the species needs to be further improved regarding some important agronomic traits. One of them is its low oil content which needs to be increased. As far as we know there is no study aiming at increasing the oil content that has been reported in this species. In order to investigate the possibility to increase the seed oil content in field cress, we have tried to introduce the *Arabidopsis WRINKLED1* (*AtWRI1*) or hemoglobin (*Hb*) genes from either *Arabidopsis thaliana* (*AtHb2*) or *Beta vulgaris* (*BvHb2*) into field cress with the seed specific expression. The hypothesis was that the oil content would be increased by overexpressing these target genes. The results showed that the oil content was indeed increased by up to 29.9, 20.2, and 25.9% in the transgenic lines expressing *AtWRI1, AtHb2*, and *BvHb2*, respectively. The seed oil composition of the transgenic lines did not significantly deviate from the seed oil composition of the wild type plants. Our results indicate that genetic modification can be used in this wild species for its fast domestication into a future economically viable oilseed and catch crop.

## Introduction

A growing world population, changing climate, and depletion of fossil oils in a near future require sustainable oil sources for a bioeconomy-based society. Plant oil has become one of the very attractive alternatives to fossil oil in the recent years as it is renewable and can be easily tailor-made for end uses using gene technology. Plant oil is widely used as food oil, in the food or chemical industries and as biodiesels. Today, a few oil crops constitute the greatest part of the world plant oil production, and the possibility to increase the oil content of these crops is limited due to various reasons. Rapeseed and oil palm already have high oil content, and the potential for further increasing the seed oil content is rather limited. An increase in the oil content of soybean would be on the expense of the protein content and is thus not alternative since protein is the most desirable component of the soybean seeds (Carlsson et al., 2011). Developing or domestication of new and high-yielding oil species with oil compositions tailored for end users would be a better choice in this context.

Field cress (*Lepidium campestre*) is a wild species with promising agronomic traits, and thus has a potential to become a future new oilseed crop. It belongs to the Brassicaceae family and is distributed all over the world (Al-Shehbaz, 1986). It has shown a high-yield potential (5 ton/ha) under field conditions with extensive weed control, and it is also winter hardy (Nilsson et al., 1998). Being a biennial species, it has the potential to function as a catch crop for reduced tillage and nutrient leaching (Merker et al., 2010). One of the major problems with field cress is the low oil content, which is around 20%. Moreover, the oil composition was not favorable as food oil. Therefore, both the oil content and composition need to be improved. Increasing the oil content by intra-specific crossing is inefficient as the genetic variation in oil content is low within the *L. campestre* species according to our preliminary studies. Genetic engineering would be a better alternative to modify the oil content and composition. Since this is still a wild species, no studies on oil content have been reported in the species so far. However, in our previous study in this species, the oil composition was altered by downregulation of two key enzymes. The oxidative and temperature stable oleic acid was increased from around 10% in the wild type (WT) to over 80% in the transgenic lines. Furthermore, the level of erucic acid was decreased from over 20% down to 0.2% (Ivarson et al., 2016).

The seed oil, mainly in the form of triacylglycerol (TAG), is largely synthesized through the Kennedy pathway where several key enzymes have been identified (Kennedy, 1961). Upregulation of these enzymes could result in alteration of seed oil content. It has been reported that both overexpression of the enzyme glycerol-3-phosphate acyltransferase (*GPAT*) and diacylglycerol acyltransferase (*DGAT*) could increase the oil content in *Arabidopsis* (Jain et al., 2000; Jako et al., 2001). However, a more efficient way is to modify expression of transcription factors that are involved in the oil biosynthesis. The most successful example in this regard is the transcription factor *WRINKLED1* (*WRI1*) (Focks and Benning, 1998; Cernac and Benning, 2004), in which a *wri1* knock out mutation resulted in a seed oil content reduction of 80%. An overexpression of a *WRI1*-like gene from *Brassica napus* in *Arabidopsis* yielded an increase in seed oil content of 10–40% in the transgenic lines (Liu et al., 2010). Overexpression of maize *WRI1* in maize resulted in transgenic lines with up to 46% increase in oil content (Shen et al., 2010). By simultaneously overexpressing *WRI1* and *DGAT1* and suppress the triacylglycerol lipase *SUGARDEPENDENT1* (*SDP1*) in *Arabidopsis*, an increase in both seed oil content and seed mass was achieved. Individual manipulations of the three genes resulted in lower increases in seed oil content and seed mass, thus an additive effect of the multigene engineering was speculated (van Erp et al., 2014). The transcription factors *B. napus LEAFY COTYLEDON1* (*BnLEC1*) and *LEC1-LIKE* (*BnL1L*) were conditionally expressed in canola, resulting in oil content increases of 2–20% without any negative influence on major agronomic traits (Tan et al., 2011).

Plant hemoglobin genes have also been proved to increase the seed oil content (Vigeolas et al., 2011). There are in principal two types of plant hemoglobins, i.e., symbiotic and non-symbiotic hemoglobins (nsHbs). The symbiotic hemoglobins (leghemoglobins) are mainly found in the root nodules of legumes that are infected by symbiotic nitrogen-fixing bacteria, and are believed to facilitate oxygen transport to the bacteria (Trevaskis et al., 1997) and at the same time buffer the level of free oxygen available in order to avoid inactivation of the oxygen-sensitive bacterial nitrogenase enzyme (Appleby et al., 1988). The nsHbs are known to be involved in nitric oxide (NO) metabolism, and possibly serve a role in signal transduction pathways of plant hormones (Hill, 2012). nsHbs are divided into mainly two classes; class 1 (nsHb1) and class 2 (nsHb2), based on phylogenetic characteristics, patterns of gene expression and their oxygen-binding capacities (Vigeolas et al., 2011). The expression of class-1 nsHb1 genes has usually been reported in organs different from those related to reproduction or reproductive stages (Nie and Hill, 1997; Trevaskis et al., 1997; Parent et al., 2008). In *Arabidopsis* and sugar beet, for instance, class-1 nsHb1 have been shown to be expressed in seeds, germinating seedlings, hypocotyls and roots (Trevaskis et al., 1997; Leiva-Eriksson et al., 2014). Differently, class-2 nsHbs genes have often been detected in reproductive organs or in processes related to such as embryogenesis and seed maturation. Thus, class-2 nsHbs have been found to be expressed in flowers of *Arabidopsis* and sugar beet (Trevaskis et al., 1997; Leiva-Eriksson et al., 2014).

Class 1-nsHbs have a high affinity for oxygen, while class 2-nsHbs show a much lower oxygen affinity. The functions of *AtHb2* is not as established as is for *AtHb1*, and due to their different characteristics their functions likely differ, but might also overlap (Spyrakis et al., 2011; Vigeolas et al., 2011; Leiva-Eriksson et al., 2014). Vigeolas et al. (2011) overexpressed endogenous *AtHb2* in developing seeds of *Arabidopsis*, which resulted in an increase in α-linolenic acid and a 40% increase in the total fatty acid content. The increase was explained to be due to an elevated energy state and sucrose content of the seeds.

In this study, the *Arabidopsis AtWRI1* and class 2-nsHb genes from *Beta vulgaris* (*BvHb2*) and *Arabidopsis thaliana* (*AtHb2*) were introduced by *Agrobacterium* mediated transformation into field cress for increasing the seed oil content.

## Materials and Methods

### Plant Material

The field cress (*L. campestre*) seeds used in this study were originally collected by late Professor Arnulf Merker in Öland, Sweden and the accession number is NO94-7.

### Surface Sterilization and Germination of Seeds

Prior to sowing, the seeds were surface sterilized in 3% calcium hypochlorite (CaCl_2_O_2_) with Tween20, with shaking for 15 min. The seeds were rinsed thoroughly with sterile water, sowed on germination medium as described by Ivarson et al. (2013) and allowed to germinate in light for 5 days.

### *In vitro* Culture Conditions

All *in vitro* cultures were maintained in a growth chamber with a day length of 16 h at 33 μmol m^-2^ s^-1^ and a temperature of 21°C and a dark period of 8 h with a temperature of 18°C. The transgenic lines and the WT plants were cultured under identical conditions, but covered with perforated plastic bags to avoid cross pollination.

### Transformation Vectors

Three different constructs were used for transformation by the *Agrobacterium* strain AGL-1: (1) *AtWRI1*-construct; (2) *AtHb2*-construct; (3) *BvHb2*-construct. The *AtWRI1* gene according to *AtWRINKLED1* (Cernac and Benning, 2004), *AtHb2* according to class-2 nsHbs from *Arabidopsis* (accession no. NM_111887.2) and *BvHb2* according to class-2 nsHbs from *B. vulgaris* (accession no. KF549982.1) were custom synthesized (Eurofins/MWG, Ebersberg, Germany or Epoch Life Signs, Inc., Missouri City, TX, USA) and then cloned into the transformation vector pBINPLUS/ARS (Belknap et al., 2008). All three target genes are under the seed specific promoter Fp1, generated from *B. napus* (Stalberg et al., 1993). After the sequence confirmation, the vectors were mobilized into the *Agrobacterium* strain AGL-1 for plant transformation, which was carried out according to the protocol by Ivarson et al. (2013).

### PCR Analysis

Regenerated shoots that were of good growth vigor were analyzed through polymerase chain reaction (PCR) analysis. Total genomic DNA was extracted from the *in vitro* grown shoots by the CTAB method (Aldrich and Cullis, 1993). Successful integration of the transgenes *nptII, AtWRI1, AtHb2*, and *BvHb2* was analyzed by PCR. The primers used for the *nptII* gene was: 5′-GCCCTGAATGAACTGCAGGACGAGGC-3′ and 5′-GCAGGCATCGCCATGGGTCACGACGA-3′, yielding a product of 411 bp, for the *AtWRI1* gene: 5′-CGGGATCCCTCATCCCCTTTTA-3′ and 5′-CGGTGGTTCTTCCACGTACT-3′ yielding a product of 1213 bp, for the *AtHb2* gene: 5′-AGACATCCCCAAATACAGCC-3′ and 5′-TGAAGACTTTAACAGCATGAGC-3′ yielding a product of 146 bp and for the *BvHb2* gene: 5′-GCAAAATATCCCAGAATACAGCC-3′ and 5′-TGGAACTTCCTCTGAATCCC-3′ yielding a product of 106 bp.

### Southern Blot Analysis

In order to further confirm the transgene integration and to determine the number of transgene copies in the transgenic lines, Southern blot analysis was performed. Approximately 20 μg of genomic DNA, extracted from *in vitro* grown shoots using the CTAB method (Aldrich and Cullis, 1993), was digested with the *Bgl*II restriction enzyme. The probes were synthesized using the same primers as for the PCR analysis in accordance with Zhu et al. (2008). The non-radioactive DIG system was used for the Southern blot hybridization (Zhu et al., 2008).

### Western Blot Analysis

Western blot analysis was conducted to identify the *AtHb2* protein expression in the transgenic lines through SDS-PAGE gel electrophoresis and immunoblotting. For protein extraction, soluble proteins were extracted from 1 mg of ground seed material in 20 μl of extraction buffer (62.5 mM Tris-HCl, pH 7.5 containing 2% SDS, 10% Glycerol, 1 mM EDTA, 5 mM dithiothreitol and 0.5% plant protease inhibitors [Sigma-Aldrich, St. Louis, MO, USA]) and centrifuged at 20.200 *g* at 4°C for 20 min. The total protein content in each sample was determined by bicinchoninic acid (BCA; Pierce Biotechnology, Inc., Rockford, IL, USA) protein assay using bovine serum albumin (BSA) as standard. For protein immunoblots, equal amount (20 μg) of extracted protein samples were separated by Bolt^TM^ 4–12% Bis-Tris Plus gels (Invitrogen, Life Technologies, Carlsbad, CA, USA) under reducing conditions and transferred to polyvinylidene fluoride (PVDF) membrane using iBlot Gel Transfer Device (Program P0) (Invitrogen, Life Technologies, Carlsbad, CA, USA). The transferred membrane was blocked with SuperBlock T20 (PBS) blocking buffer (Thermo Fisher Scientific, Inc., Waltham, MA, USA) at room temperature with rocking for 1 h, followed by incubation in the anti-AtHb2 primary antibody (1:1000; Agrisera) in the SuperBlock T20 (PBS) blocking buffer for 1 h at 22°C. Then the membrane was washed 3 times with PBS Tween^®^-20 (Thermo Fisher Scientific, Inc., Waltham, MA, USA) for 5 min each and probed with Novex^®^ anti-rabbit secondary antibody conjugated with horseradish peroxidase at a 1:2000 dilution in SuperBlock T20 (PBS) blocking buffer. Detection was carried out with Novex^®^ ECL chemiluminescent substrate reagent kit (#WP20005; Invitrogen). MagicMark XP (#LC5602; Invitrogen) Western Protein Standard was used to determine relative molecular weights.

### Quantitative Real Time PCR (qRT-PCR) analysis

Immature pods were collected 28 days after flowering and total RNA was extracted using the RNeasy Plant Mini Kit (Qiagen, Hilden, Germany). Apart from some minor alterations, the manufacturer’s protocol was followed for RNA extraction. The extracted RNA was treated with DNase (TURBO DNA-free (Ambion, Austin, TX, USA) to remove genomic DNA. First strand cDNA was synthesized from 1000 ng RNA by using Superscript III First-Strand Synthesis Supermix for qRT-PCR (Invitrogen, Life Technologies, Carlsbad, CA, USA) in a 20 μl reaction. The obtained cDNA was diluted five times and 3 μl was used for each 20 μl qRT-PCR reaction using BIO-RAD C1000 Thermal Cycler, CFX 96 Real-Time System (Foster City, CA, USA) with BIO-RAD iQ SYBR Green Supermix (Bio-Rad, Hercules, CA, USA). The PCR program used was 95°C for 10 min followed by 40 cycles of 95°C for 15 s, 63°C for 30 s and 72°C for 30 s. To confirm product specificity, a melt curve analysis was included in the analysis. The primer pairs for the *AtWRI1, AtHb2*, and *BvHb2* genes (Supplementary Table S1) were chosen among several pairs tested for their efficiency and specificity. The *TIP41-like* reference gene (Supplementary Table S1) was used as a reliable reference in this study as reported by Lenser and Theissen (2013), Muhlhausen et al. (2013), and Ivarson et al. (2016). For each line, three biological replicates and three technical replicates were conducted.

### Plant Growth Conditions and Management

Confirmed transgenic lines together with the WT plants were *in vitro* vernalized at 4°C for 8 weeks in a growth chamber with a light intensity of 30 μmol m^-2^ s^-1^ before they were planted in the biotron, in which the growing conditions were 16-h photoperiod with 250 μmol m^-2^ s^-1^ light intensity, 21/18°C temperature (day/night) and 60% humidity. The plants were fertilized every 8 weeks with long-lasting granules (N: P: K = 21: 3: 10) and watered regularly. Mature seeds were harvested and dried before threshing. The seeds were then stored at 4°C until further analysis.

### Oil Content Analysis

Triplicates of pooled samples of three seeds per replicate were weighed and placed in glass tubes with addition of 1 ml 0.15 M acetic acid and 3.75 ml MeOH:CHCl_3_ (2:1). The samples were then homogenized by IKA^®^ T18 basic (ULTRA TURRAX^®^), followed by addition of 1.25 ml CHCl_3_ and 0.9 ml H_2_O. Then the samples were vortexed and centrifuged for 2 min at 3000 rpm before 200 μl of the chloroform phase was pipetted to a new tube. The tubes were then allowed to dry completely under nitrogen before 100 μl hexane, 100 nmols of internal standard 17:0 (in methanol) and 2 ml methylation solution (2% H_2_SO_4_ in methanol) was added to the tubes. The tubes were left to methylate at 90°C for 1 h. After methylation, the samples were left to cool down before 1 ml H_2_O and 600 μl hexane was added. The samples were vortexed and centrifuged for 2 min at 2000 rpm and finally, 200 μl of the hexane phase was transferred to a GC vial with an insert. The samples were analyzed on an Agilent (model 7890A) gas chromatograph with a WCOT Fused Silica CP-Wax 58 column and a FID detector (Agilent technologies, Santa Clara, CA, USA).

### Experimental Design and Statistical Analysis

The seed oil content was analyzed in triplicate samples with three seeds per replicate from the WT plants and the transgenic lines, respectively. In total, 10 WT plants from T_1_, 12 WT plants from T_2_ and 17 WT plants from T_3_ were analyzed. Each transgenic line corresponds to one plant. The seed oil content was calculated based on the peak areas in the chromatograms from the GC analysis. The mean seed oil content and the standard deviation were calculated. The data was analyzed by Fisher pairwise comparisons with a significance level of 95%, using the Minitab program.

The seed weight was analyzed from triplicate samples with 100 seeds per replicate from a WT plant and the transgenic lines. The mean seed weight and the standard deviation were calculated. The seed weights were compared using Fisher pairwise comparisons with a significance level of 95% in the Minitab program.

The seed oil composition of triplicate samples with three seeds per replicate was calculated based on the peak areas in the chromatograms from the GC analysis. The mean and standard deviation were decided and the data was analyzed with Tukey pairwise comparisons with a significance level of 95% using Minitab.

The gene expression was analyzed in three biological replicates per WT plant or transgenic line. Three technical replicates per biological replicate were analyzed in the qRT-PCR analysis. The relative expression level was calculated and the data was analyzed with Tukey pairwise comparisons with a significance level of 95%, using the Minitab program.

## Results

### Molecular Analysis of Transgenic Lines

Polymerase chain reaction analysis on the genomic DNA from putative transgenic lines showed successful integration of the *AtWRI1* gene in 19 lines, *AtHb2* in 20 lines and *BvHb2* in 23 lines. All the transgenic lines harbor also the *nptII* gene for selection of transformants (**Supplementary Figure S1**).

**FIGURE 1:**
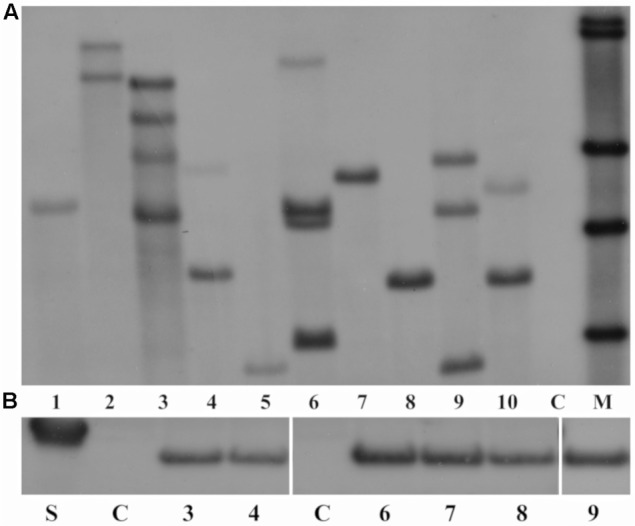
**(A)** Results of the Southern blot analysis of the T_1_ transgenic lines 1–10 (Lanes 1–10) and wild type (WT) (lane 11) of field cress. Lanes 1–3 = transgenic lines overexpressing *AtWRI1*, lanes 4–5 = transgenic lines overexpressing *AtHb2* and lanes 6–10 = transgenic lines overexpressing *BvHb2.* C, WT. M, molecular markers. The DNA was digested with the *Bgl*II restriction enzyme and hybridized with the DIG-labeled *nptII* probe. **(B)** Gel image of Western blot analysis of AtHb2 lines from T_1_ and T_3_ generation. Lane 1 = marker (20 kDa), Lane 2 = WT from T_1_ (C), Lanes 3 and 4 = T_1_ *AtHb2* lines 6 and 5, Lane 5 = WT from T_3_ (C), Lanes 6–9 = T_3_ AtHb2 lines 5-1-1 – 5-1-3 and 6-1-1.

Southern blot analysis showed that hybridization with the *nptII* probe gave rise to clear bands, indicating a stable integration of the T-DNA into the field cress genome. As shown in **Figure 1A**, the copy number of the *nptII* gene ranged from 1 to 4, with 4 out of 10 analyzed lines showing single copy integration of the transgene. The *AtWRI1* overexpressing line 2 (lane 1, **Figure 1A**) and the *BvHb2* overexpressing line 5 (lane 8, **Figure 1A**) were two of the lines that were further analyzed up to T_4_.

All *AtHb2*-expressing lines showed protein expression of the *AtHb2* gene with varying band intensity in both first and third generations, while no AtHb2 protein expression was found in the WT seeds in both generations (**Figure 1B**). This indicates the *AtHb2* gene was stably expressed in the subsequent generations.

### Plant Performance and Seed Oil Content

When grown in biotron, the transgenic plants did not show any phenotypic deviations from the WT plants, in terms of plant height, flowering time, seed setting and seed development.

To evaluate the influence of the expression of the transgenes on seed oil content, total seed fatty acid content was analyzed in mature seeds using gas chromatography analysis. The lines exhibiting the highest levels of oil contents in T_1_ were further evaluated in the subsequent generations up to T_4_. Since the oil content is easily affected by environmental factors, all transgenic lines including the WT were grown under identical conditions in the biotron. To simplify the table, only the lines showing the increased oil content are presented in **Table 1**. For all three constructs, transgenic lines with significantly increased seed oil content were found in T_1_ (**Table 1**). For the lines overexpressing the *AtWRI1* gene, two transgenic lines showed greater oil content increase than the other lines in T_1_. Of the two lines, one had an oil content of 24.3% compared to 21.8% in the WT, generating an oil increase of 11.4%. Another line showed a greater oil increase of 17.9%, with the oil content of 25.7%. In T_2_, the two lines showing highest oil contents had an increased oil content of 18.1 and 29.9%, respectively compared to the WT. The oil content of the T_3_ generation was generally lower for both WT and *AtWRI1* lines compared to the T_1_ and T_2_ generations. The two lines with greatest increase in seed oil content had the oil content of 22.4 and 23.6%, respectively compared to 19.9% for the WT, generating an oil increase of 12.4 and 18.3%, respectively (**Table 1**).

**Table 1:** Seed oil content and percentage of increase in seed oil content in three generations of transgenic lines of field cress overexpressing *AtWRI1, AtHb2*, and *BvHb2* genes, respectively.

*AtWRI1*			*AtHb2*			*BvHb2*		
Gen./line	Oil content (%)^∗^	Increase (%)	Gen./line	Oil content (%)^∗^	Increase (%)	Gen./line	Oil content (%)^∗^	Increase (%)
**T1**			**T1**			**T1**		
WT^∗∗^	21.8 ± 0.7 c		WT	21.8 ± 0.7 c		WT	21.8 ± 0.7 c	
1	25.7 ± 0.4 a	17.9	5	24.0 ± 0.5 b	10.4	9	27.4 ± 0.2 a	25.9
2	24.3 ± 1.0 ab	11.4	6	25.9 ± 0.6 a	18.9	10	25.9 ± 2.0 ab	18.9
3	23.8 ± 0.9 b	9.2	7	24.7 ± 0.8 ab	13.4	11	27.0 ± 0.3 ab	23.9
4	23.4 ± 1.1 b	7.3	8	24.4 ± 0.4 b	11.7	12	25.1 ± 1.0 b	15.3
**T2**			**T2**			**T2**		
WT^∗∗^	21.4 ± 1.7 b		WT	21.4 ± 1.7 b		WT	21.4 ± 1.7 a	
1-1	25.3 ± 1.5 a	18.1	5-1	24.4 ± 1.7 a	14.1	9-1	24.0 ± 1.0 a	12.3
2-1	27.8 ± 4.2 a	29.9	6-1	23.1 ± 1.5 a	7.8	10-1	21.7 ± 2.0 a	1.4
**T3**			**T3**			**T3**		
WT^∗∗^	19.9 ± 1.3 c		WT	19.9 ± 1.3 b		WT	19.9 ± 1.3 c	
1-1-1	20.4 ± 2.7 bc	2.3	5-1-1	23.6 ± 2.4 a	18.4	9-1-1	22.1 ± 0.3 ab	10.9
1-1-2	23.6 ± 1.0 a	18.3	5-1-2	23.9 ± 0.7 a	20.2	9-1-2	20.5 ± 0.8 bc	3
1-1-3	22.4 ± 0.7 ab	12.4	5-1-3	23.2 ± 0.9 a	16.4	9-1-3	23.2 ± 0.4 a	16.7
2-1-1	20.2 ± 0.8 bc	1.2	6-1-1	20.1 ± 1.0 b	0.9	9-1-4	22.6 ± 0.9 a	13.7

*Results represent mean ± SD from three replicates of pooled seeds per transgenic line, 10 WT plants from T_1_, 12 WT plants from T_2_, and 17 WT plants from T_3_*

*^∗^Lines that do not share the same letter within each generation are significantly different at *p* = 0.05. Data was analyzed with Fisher pairwise comparisons using Minitab program. ^∗∗^The oil content of WT plants ranged from 20.1 to 22.7% in T_1_, 18.6 to 24.5% in T_2_, and 16.9 to 22.1% in T_3_.*

For the transgenic lines harboring the *AtHb2* gene, four lines showed significantly increased oil contents of 10.4, 11.7, 13.4, and 18.9%, respectively, compared to the WT in T_1_. In T_2_, lines originating from lines 5 and 6 in T_1_ showed a significant increase in oil content. However, the increase was lower in T_2_ compared to T_1_, with an increase of 7.8 and 14.1%, respectively for the two lines. The oil content in T_3_ was generally lower for both transgenic lines and WT; however, the increase in oil content was higher in T_3_ compared with T_1_ and T_2_. The three individual seed lines showing the highest oil increase ranged from 16.4 to 20.2% (**Table 1**).

Transgenic lines overexpressing the *BvHb2* gene showed the higher oil increases in T_1_ compared to the lines transformed with the other two constructs. The best performing line had an oil content of 27.4% compared to 21.8% in WT, generating an oil increase of 25.9%. The other three lines had the oil content increased by 15.3, 18.9, and 23.9% respectively. However, the same high level of increase was not detected in the subsequent generations. The highest increase found in T_2_ was 12.3 and 16.7% in T_3_.

### Seed Weight

Seed weight differs in some cases, for instance, the seed weight of the third generation transgenic lines overexpressing *AtWRI1* (1-1-3 and 2-1-1) was significantly higher (increased by 3.5 and 12.4%, respectively), while two of the lines (1-1-1 and 1-1-2) were significantly lower (decreased by 8.4 and 7.6%, respectively) than the WT (**Table 2**). Regarding the *AtHb2* overexpressing lines, one line in the third generation (5-1-3) showed a modest, but significant increase in seed weight by 3.6%, while the other three lines (5-1-1, 5-1-2, and 6-1-1) showed a significantly lower seed weight compared to the WT. A significant increase in seed weight was also seen in one line (9-1-2) of third generation *BvHb2*-overexpressing plants, while two of the remaining lines showed a decrease (**Table 2**).

**Table 2:** Seed weight (mean ± SD^∗^ from three replicates per line) in three generations of field cress lines overexpressing *AtWRI1, AtHb2*, and *BvHb2*, respectively.

*AtWRI1*	mg/100 seeds^∗^	*AtHb2*	mg/100 seeds^∗^	*BvHb2*	mg/100 seeds^∗^
**T1**		**T1**		**T1**	
WT	305.1 ± 2.1 a	WT	305.1 ± 2.1 a	WT	305.1 ± 2.1 a
1	310.3 ± 4.9 a	5	249.8 ± 0.2 c	9	300.9 ± 3.2 a
2	308.9 ± 2.1 a	6	301.2 ± 1.4 b	10	268.2 ± 4.3 b
**T2**		**T2**		**T2**	
WT	278.3 ± 1.0 a	WT	278.3 ± 1.0 a	WT	278.3 ± 1.0 a
1-1	258.4 ± 1.4 b	5-1	266.1 ± 5.2 b	9-1	263.3 ± 2.5 b
2-1	NA^∗∗^	6-1	259.9 ± 2.4 b	10-1	263.5 ± 5.4 b
**T3**		**T3**		**T3**	
WT	301.9 ± 3.3 c	WT	301.9 ± 3.3 b	WT	301.9 ± 3.3 b
1-1-1	276.4 ± 9.2 d	5-1-1	253.1 ± 1.4 d	9-1-1	303.2 ± 1.6 b
1-1-2	279.0 ± 1.8 d	5-1-2	289.7 ± 1.2 c	9-1-2	309.9 ± 1.9 a
1-1-3	312.6 ± 0.4 b	5-1-3	312.8 ± 1.1 a	9-1-3	289.7 ± 1.8 c
2-1-1	339.2 ± 1.4 a	6-1-1	295.4 ± 2.9 c	9-1-4	285.9 ± 1.5 c

*^∗^Lines that do not share the same letter with each generation are significantly different at *p* = 0.05. Data was analyzed with Fisher pairwise comparisons using Minitab program.*

*^∗∗^Not available due to insufficient number of seeds.*

**FIGURE 2:**
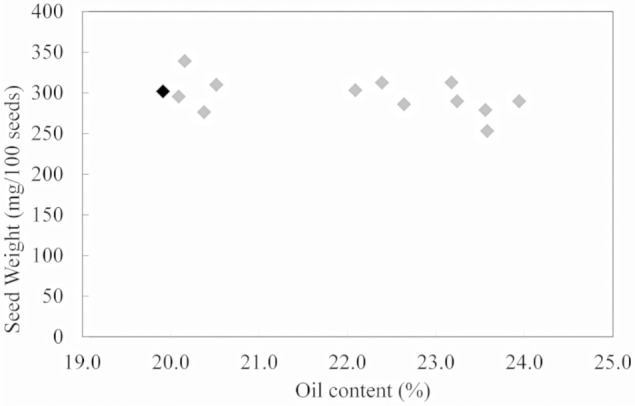
**Correlation analysis between seed weight and seed oil content in WT (black) and transgenic lines of field cress (gray)**.

Further analysis on changes in seed oil content and seed weight showed no significant relation between these two alterations in transgenic lines of field cress (**Figure 2**).

### Seed Oil Composition

The seed oil composition in the transgenic lines was in general not different from the oil composition of the WT. Minor deviations were found, for instance, the *AtWRI1-*expressing line 1-1-2 (**Table 3**) showed a significant decrease in the levels of 18:1, 18:2, 20:1, and 22:1, however, this change was not seen among the other *AtWRI1-*expressing lines. The only deviation found among the *AtHb2*-overexpressing lines was a significant decrease in 22:1 in line 5-1-2. One *BvHb2*-overexpressing lines (9-1-2) had a significantly lower level of 18:2, 20:1 and 22:1 fatty acids, and a second line (9-1-1) had significantly lower levels of 18:3 and 22:1 fatty acids, however, no clear trend was seen among the lines.

**Table 3:** Seed oil composition (%, mean ± SD^∗^ from three replicates per line) in T3 transgenic lines overexpressing *AtWRI1, AtHb2*, and *BvHb2* genes, respectively, and wild type of field cress.

Line	18:1	18:2	18:3	20:1	22:1	24:0
***AtWRI1***						
WT	11.8 ± 0.7 a	6.7 ± 0.2 a	28.7 ± 0.6 a	4.1 ± 0.2 b	26.5 ± 0.6 a	2.0 ± 0.5 a
1-1-1	10.7 ± 0.9 ab	6.2 ± 0.2 ab	27.3 ± 0.5 a	3.6 ± 0.5 b	23.4 ± 0.2 b	2.1 ± 0.2 a
1-1-2	9.4 ± 0.3 b	5.6 ± 0.6 b	27.6 ± 1.5 a	3.2 ± 0.3 a	23.3 ± 0.8 b	2.8 ± 2.3 a
1-1-3	12.0 ± 0.5 a	6.1 ± 0.2 ab	27.4 ± 0.2 a	3.9 ± 0.2 b	23.8 ± 0.2 b	1.8 ± 1.0 a
2-1-1	10.2 ± 0.5 ab	5.9 ± 0.4 ab	28.8 ± 0.7 a	3.4 ± 0.1 b	25.4 ± 0.1 a	1.9 ± 0.2 a
***AtHb2***						
WT	11.8 ± 0.7 ab	6.7 ± 0.2 ab	28.7 ± 0.6 ab	4.1 ± 0.2 ab	26.5 ± 0.6 a	2.0 ± 0.5 a
5-1-1	14.0 ± 1.5 a	6.3 ± 0.1 b	27.4 ± 1.4 ab	4.6 ± 0.4 a	26.4 ± 0.6 a	1.4 ± 1.2 a
5-1-2	14.1 ± 0.4 a	6.9 ± 0.3 ab	26.5 ± 1.3 b	4.6 ± 0.1 a	20.9 ± 1.2 b	1.2 ± 0.9 a
5-1-3	12.6 ± 0.7 ab	6.7 ± 0.2 ab	30.0 ± 0.8 a	4.7 ± 0.1 a	26.5 ± 0.1 a	1.2 ± 0.8 a
6-1-1	11.0 ± 0.5 b	7.3 ± 0.3 a	29.7 ± 0.3 a	4.0 ± 0.2 ab	27.2 ± 0.3 a	1.8 ± 0.4 a
***BvHb2***						
WT	11.8 ± 0.7 ab	6.7 ± 0.2 a	28.7 ± 0.6 a	4.1 ± 0.2 a	26.5 ± 0.6 a	2.0 ± 0.5 a
9-1-1	12.6 ± 0.4 a	6.4 ± 0.1 ab	26.9 ± 0.4 b	3.9 ± 0.1 ab	20.7 ± 0.7 b	1.9 ± 0.0 a
9-1-2	10.9 ± 0.2 b	5.9 ± 0.2 b	27.8 ± 0.3 ab	3.3 ± 0.1 b	20.0 ± 1.3 b	1.9 ± 0.3 a
9-1-3	11.1 ± 0.0 b	6.4 ± 0.1 ab	28.4 ± 1.0 ab	4.0 ± 0.1 a	24.8 ± 0.4 a	1.7 ± 0.1 a
9-1-4	11.9 ± 0.9 ab	6.3 ± 0.2 ab	28.7 ± 0.5 a	4.3 ± 0.4 a	26.1 ± 0.4 a	1.5 ± 0.1 a

*^∗^Lines that do not share the same letter within each generation are significantly different at *p* = 0.05. Data was analyzed with Tukey pairwise comparisons using Minitab program.*

### Quantitative Real Time PCR (qRT-PCR) Analysis

**FIGURE 3:**
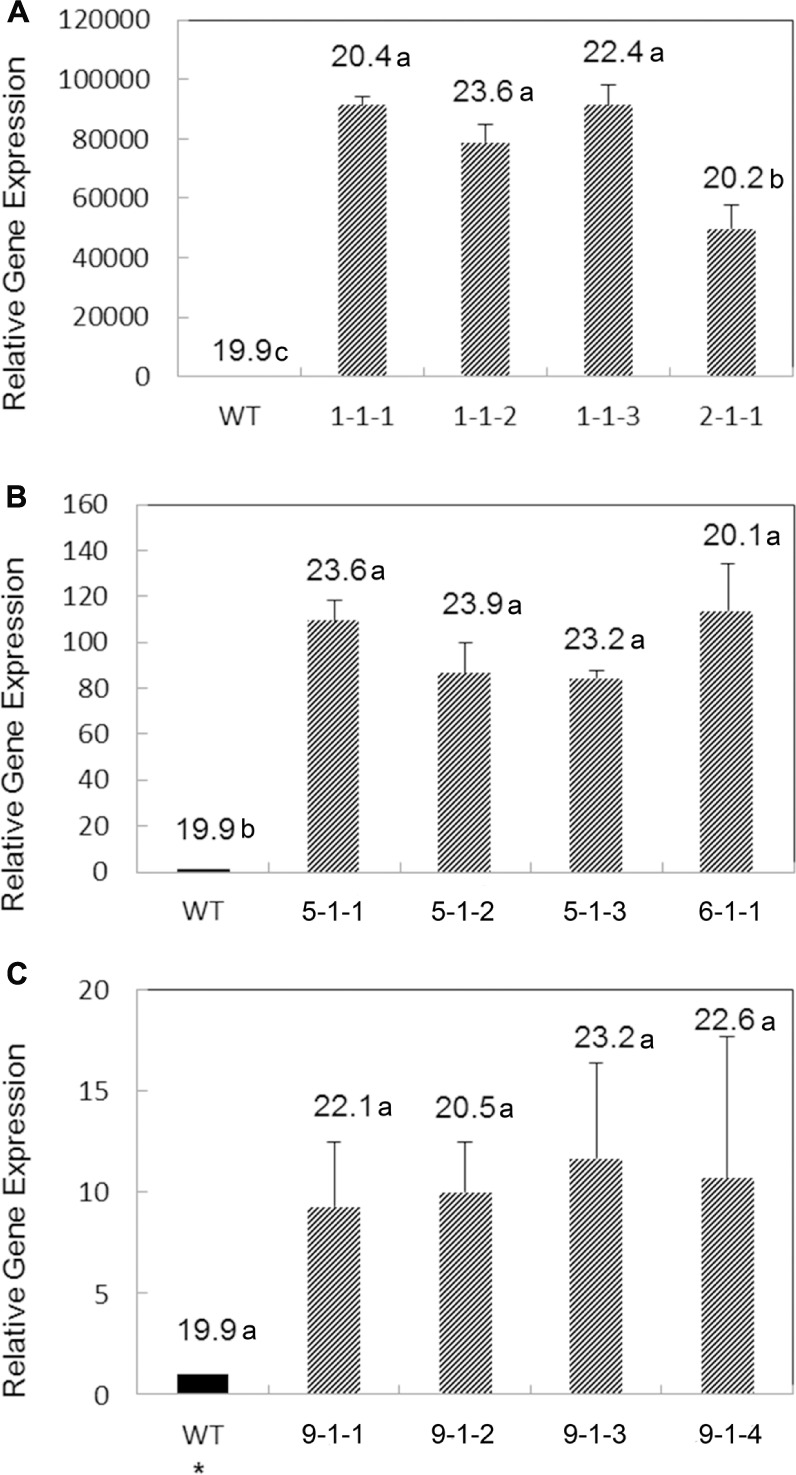
**Relative gene expression levels of the *AtWRI1* (A)**, *AtHb2* **(B)**, and *BvHb2* **(C)** genes in immature seeds of T_3_ transgenic lines in comparison with the WT. Results are means from three biological replicates with three technical replicates per biological replicate. Error bars represent standard deviation (SD). Figures above the bars represent the mean oil content (%) in the respective lines. Bars that do not share the same letter are significantly different at *p* = 0.05. Data was analyzed with Tukey pairwise comparison using Minitab program. ^∗^Estimated value since no expression of *BvHb2* in WT, which was generated by dividing the lowest expression value of a transgenic line by 10.

Transgene expression of five transgenic T_3_ lines from each construct was analyzed by qRT-PCR. In order to compare transgene expression levels of *AtWRI1, AtHb2* and *BvHb2*, transgenic lines with both moderate and higher increase in oil content were chosen for analysis. In the *AtWRI1* overexpressing lines, overexpression of *AtWRI1* was found in all transgenic lines, with a relative expression level up to over 90.000-fold higher than in the WT (**Figure 3**). The line having the lowest relative *AtWRI1* expression level corresponded to the lowest oil content among the lines analyzed, indicating the gene expression level of the transgene positively correlated with the seed oil content.

The relative expression level of *AtHb2* was higher in the transgenic lines compared to the WT, with a fold-increase ranging between 84 and 114.

A much lower transgene expression level was detected in the *BvHb2* overexpressing lines among the three types of transgenic lines. The transgenic lines showed a relative expression level increased by a fold-change of 9.3 to 13.7, with a positive correlation between oil content and gene expression level.

## Discussion

As sustainable resources, plants have a great potential to provide tailor-made oil qualities for a bioeconomy-based society. However, the amount of plant oils produced and the variation of oil qualities available today are very limited, mainly due to the limited oil crop species available in commercial production, which are restricted to certain climate conditions. The need for developing new and high yielding oil crops with novel and favorable oil compositions, which can be adapted to certain climate conditions, such as cold regions or marginal lands is thus crucial. Owing to its favorable agronomic characteristics, the wild field cress has potential to be domesticated into a novel oilseed crop. As it is biennial, it can be cultivated as a catch crop with less need for tillage and reduced nutrient leaching, thus having environmental benefits. Also, due to its high winter hardiness, it can be cultivated in regions where winter is harsh.

Several attempts to alter the oil content of various crops have been made, both by upregulation of the key enzymes in the oil biosynthesis (Jako et al., 2001; Lardizabal et al., 2008; Liu et al., 2015) and regulations of some important transcription factors involved in the oil biosynthesis (Liu et al., 2010; Shen et al., 2010; An and Suh, 2015). For instance, overexpression of *DGAT* in *Arabidopsis* yielded seed oil content increases of between 9 and 12% on a dry weight basis (Jako et al., 2001), while the seed oil increases ranging between 10 and 40% was generated by *BnWRI1-*overexpression in *Arabidopsis* (Liu et al., 2010).

In this study, *Arabidopsis WRI1* gene and nsHb genes from *Arabidopsis* and sugar beet were individually expressed under the seed specific promoter in field cress to investigate whether overexpression of these genes could increase the seed oil content in the target wild species. Our results showed that overexpression of the *AtWRI1, AtHb2*, and *BvHb2* gene could increase the oil content up to 29.9, 20.2, and 25.9%, respectively in field cress. A similar increase has been demonstrated in camelina, where a seed-specific overexpression of *AtWRI1* yielded a 14% increase in total seed oil content (An and Suh, 2015). An even higher increase has been shown in maize where *ZmWRI1*-overexpression generated an average increase in seed oil content of 30.6% (Shen et al., 2010). The 20.2% increase in seed oil content of *AtHb2-*overexpressing lines of field cress achieved in this study is lower compared to the increase generated in a study by Vigeolas et al. (2011), in which overexpression of *AtHb2* in *Arabidopsis* led to a 40% increase in fatty acid content in *Arabidopsis*. This is probably due to the different species studied.

In T_1_, the highest increases in the oil content were found in the lines overexpressing the *BvHb2* gene. However, the high increase was not maintained in the subsequent generations, indicating likely an unstable expression of the transgene. This can be reflected in the qRT-PCR result where it showed a much lower expression of the *BvHb2* gene, compared to these of the *AtWRI1* and *AtHb2* genes in other transgenic lines.

The phenotypic performance did not differ between the transgenic plants and the WT plants under biotron conditions, namely no deviations in flowering time, seed setting or seed development were found in this study. This is in accordance with the study where *B. napus WRI1* was overexpressed in *Arabidopsis*, leading to seed oil content increases of 10–40% (Liu et al., 2010) and the study by An and Suh (2015) mentioned above.

Seed weight increases have been reported in transgenic *Arabidopsis* lines expressing *BnWRI1* (Liu et al., 2010), and in *AtWRI1-*expressing lines of camelina (An and Suh, 2015). In this study, significant increases in seed weight were also observed in two of the third generation field cress lines overexpressing *AtWRI1*. However, the seed weights of two of the remaining T_3_ *AtWRI1*-expressing lines were significantly lower compared to the WT. The *AtHb2*- and *BvHb2-*expressing lines both had one T_3_ generation line with a significantly higher seed weight compared to the WT, while all the remaining T_3_ lines showed significant decreases or no differences in seed weight. No correlation between seed weight and seed oil content is seen in this study since the lines with the highest increases in seed oil content showed both increases and decreases in seed weight in comparison to the WT. The different degrees of increase in seed weight by the same homologous gene shown in different studies might be partially due to the position effect of transgene integration in the genome. It may also indicate a complicated relationship between oil increase and seed weight, as shown by Xu et al. (2008) where seed-specific expression of *Tropaeolum majus diacylglycerol acyltransferase 1* (*DGAT1*) resulted in increased seed weight when expressed in *Arabidopsis* but not in *B. napus*. Vigeolas et al. (2011) did not report on any change in seed weight in *AtHb2*-overexpressing lines of *Arabidopsis.*

The seed oil composition of *AtWRI1*-expressing lines was not significantly different from the oil composition of the WT in this study. This is in line with the result reported by Hu et al. (2013) where the oil content was not correlated with fatty acid composition by comparing seven lines in rapeseed. However, the effects on oil composition of *WRI1* overexpression reported in other studies vary depending on the origin of the *WRI1* gene and the target species. For instance, in camelina, overexpression of *AtWRI1* generated a seed oil composition with decreased levels of oleic acid (18:1) and eicosenoic acid (20:1), and increased levels of linoleic acid (18:2) and linolenic acid (18:3) (An and Suh, 2015). When *AtWRI1* was infiltrated into *Nicotiana benthamiana*, a decrease in stearic acid (18:0) and 18:2 and an increase in 18:3 were found (Vanhercke et al., 2013), while no significant changes in oil composition was detected in *ZmWRI1*-overexpressing seeds of maize (Shen et al., 2010) or *AtWRI1*-overexpressing seeds of *Arabidopsis* (Cernac and Benning, 2004; van Erp et al., 2014). These results may suggest a difference in the enzyme activity depending on origin of the transgene and the transgene recipient as well as the position effect of transgene integration into the host genome.

In the study by Vigeolas et al. (2011), *AtHb2* overexpression in *Arabidopsis* caused a change in the oil composition of the transgenic lines in comparison to the WT. The level of 20:1, which is a marker for storage TAG formation in *Arabidopsis* (Lemieux et al., 1990), was increased by 20–60%. Also, increases in 18:2 and 18:3 and the 18:2/18:1 and 18:3/18:2 ratios were seen. However, in the present study, neither *AtHb2-* nor *BvHb2-*overexpression resulted in any significant alterations in the seed oil composition of the transgenic field cress lines compared to the WT. This may also be species-dependent.

To further increase the seed oil content in field cress, a trial incorporating multigene engineering with a push-pull-protect-approach (Vanhercke et al., 2013) would be interesting to test in the future. Synergistic effects on seed oil content in *Arabidopsis* have also been reported by van Erp et al. (2014) when seed specific co-expression of *WRI1* (push) and *DGAT1* (pull) was combined with a mutation or downregulation of the *SUGAR-DEPENDENT1* lipase gene. Furthermore, to combine the high-oleic acid field cress lines generated in our previous study as stated above with an increase in the seed oil content would be beneficial for food processing or industrial applications since the oil would be more stable in high temperatures, and thus have a longer shelf-life.

## Conclusion

We have developed transgenic lines of field cress with up to 29.9% increase in seed oil content by overexpressing three different target genes. Thus, this study shows the potential for the wild species *L. campestre* to be domesticated into a future high-yielding oil- and catch crop.

## Author Contributions

EI participated in the design of the experiment, performed the majority of the experimental work, AA performed some experimental work, NL-E prepared the hemoglobin transformation vectors and participated in writing of the manuscript, SK performed the protein analysis and participated in writing of the manuscript, LB participated in writing of the manuscript and L-HZ designed and coordinated the project and prepared the *AtWRI1* construct. EI and L-HZ wrote the manuscript.

## Conflict of Interest Statement

The authors declare that the research was conducted in the absence of any commercial or financial relationships that could be construed as a potential conflict of interest.

## Supplementary Material

FIGURE S1 **Partial PCR results of the transgenic lines of field cress.** From left, lanes 1–5 = *AtWRI1* gene, lane 6 = plasmid DNA (+), lane 7 = wild type (WT) field cress (–), lane 8 = blank (B), lanes 9–13 = *AtHb2* gene, lane 14 = plasmid DNA (+), lane 15 = WT field cress, lane 16 = blank (B), lanes 17–20 = *BvHb2* gene, lane 21 = plasmid DNA (+), lane 22 = WT field cress (-), lane 23 = blank (B) and lane 24 = molecular markers.
