# Supplementary material for: Effects of Overexpression of WRI1 and Hemoglobin Genes on the Seed Oil Content of Lepidium campestre
Source: Front Plant Sci. 2017 Jan 9;7:2032. doi: 10.3389/fpls.2016.02032 (PMC5220066; doi:10.3389/fpls.2016.02032)
Supplement: Supplementary file 2 [file Table_1.PDF]

Supplementary table 1 Sequences of the primers used for qRT-PCR

Gene	*Forward primer (5'-3')*	*Reverse primer (5'-3')*	*Amplicon length (bp)*
LcTIP41-like	GCTTATGAGATTGAGAGAGACGAGAA	GGATACCCTTTCGCAGATAGAGAC	122
AtWRI1	AGCAGAGCAACAAGAAGCAG	AAGCCAGCTCATTGTTGTCC	122
AtHb2	TTCGAGGTGGTGAAAGAAGC	GTTTCATCTCGGTCTTGATGGC	133
BvHb2	GCAAGTGGTGACAAATGGAGTG	TTGATGGCTGCAGCTAAGTG	77
